# Production of Cellulose Nanoparticles from Cashew Apple Bagasse by Sequential Enzymatic Hydrolysis with an Ultrasonic Process and Its Application in Biofilm Packaging

**DOI:** 10.1021/acsomega.4c08702

**Published:** 2024-12-13

**Authors:** Layanne Guedes Silva de Araújo, Tigressa Helena Soares Rodrigues, Erick Rafael Dias Rates, Luciana Magalhães Rebelo Alencar, Morsyleide de Freitas Rosa, Maria Valderez Ponte Rocha

**Affiliations:** † Department of Chemical Engineering, Bioengineering and Biomass Valorization Laboratory, Federal University of Ceará, Fortaleza, Ceará 60020-181, Brazil; ‡ Exact Sciences and Technology Center, Chemistry Course, State University of Acaraú Valley, Sobral, Ceará 62040-370, Brazil; § Department of Physics, Laboratory of Biophysics and Nanosystems, Federal University of Maranhão, São Luís, Maranhão 65080-805, Brazil; ∥ Embrapa Tropical Agroindustry, Rua Dra Sara Mesquita 2270, Planalto do Pici, CEP 60511-110 Fortaleza, CE, Brazil

## Abstract

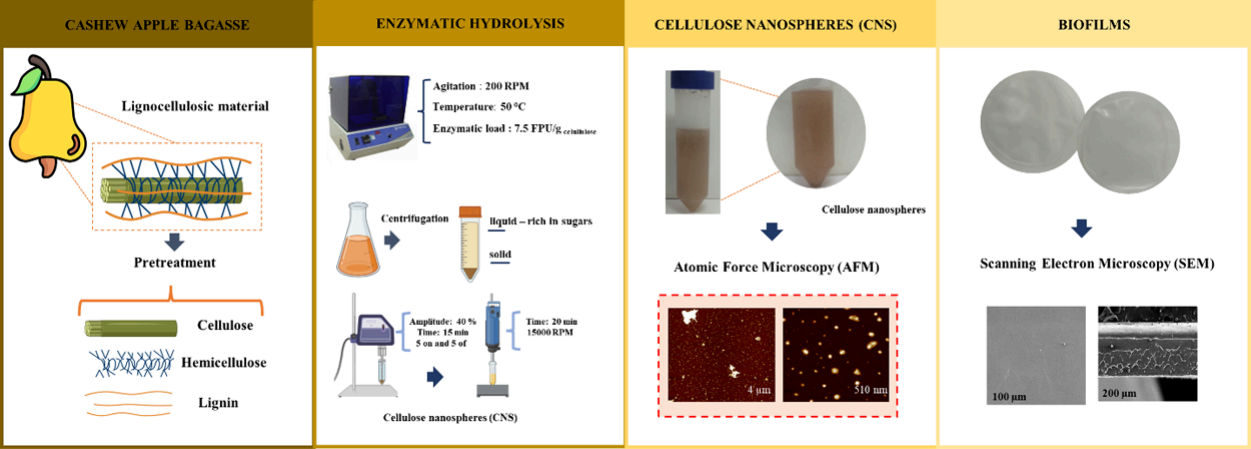

Cellulose nanostructures obtained from lignocellulosic biomass via enzymatic processes may offer advantages in terms of material properties and processing sustainability. Thus, in this study, cellulose nanoparticles with a spherical morphology were produced through the enzymatic hydrolysis of cashew apple bagasse (CAB). CAB was previously subjected to alkaline and acid-alkali pretreatment, and the pretreated solids were labeled as CAB-PTA and CAB-PT-HA, respectively. The enzymatic hydrolysis was carried out using two different enzymatic loadings (7.5 and 12 FPU/g_cellulose_) of the *Trichoderma reesei* cellulase complex, and the formation of nanostructures occurred only at 7.5 FPU/g_cellulose_. The results indicated the production of nanocellulose using only CAB-PT-HA as the precursor, obtaining nanosphere structures with a yield of 65.1 ± 2.9% and a diameter range of 57.26–220.66 nm. The nanocellulose showed good thermal and colloidal stability and was subsequently used for biofilm production. Biofilms were prepared using different percentages of nanocellulose (5 and 7% w/v), and they showed a greater water retention capacity and higher biodegradability compared to the control film, indicating potential for application in food packaging and cosmetic masks. Thus, it highlights the potential for developing new biodegradable plastics incorporated with nanocellulose obtained from CAB through a more sustainable process.

## 1 Introduction

One of the major challenges faced by the present world is finding an effective method to tackle the environmental problems caused by nondegradable plastic waste. Plastics are highly valued for their functionality, but their accumulation negatively impacts human existence, wildlife, and the entire ecosystem.^1,2^ Consequently, research is being conducted to develop biodegradable polymers.^3−5^ These biomaterials can be economically produced from bioderived monomers due to their abundant availability, cost-effectiveness, high specific strength, as well as mechanical and barrier properties.^1^ Therefore, there is a significant demand for materials sourced from nature for Bioplastic production, such as lignocellulosic waste.^6,7^

Lignocellulosic biomass is renewable and stands out as a promising feedstock to replace fossil resources in future biorefineries. However, for biorefineries to be economically viable, it is essential to integrate large-scale biofuel production with the generation of other high-value products,^7,8^ such as ethanol,^9^ Bioplastic,^10^ and nanocelluloses.^11,12^

Nanocellulose (NC) offers a wealth of possibilities for surface modifications and boasts high aspect ratios, excellent mechanical properties, and notable crystallinity due to its nanostructure. This versatility enables its application across numerous sectors^13^ and has driven research into a diverse array of products, including nanocomposites, gels, aerogels, viscosity modifiers, films, barrier layers, fibers, foams, and filtering membranes.^14,15^ Additionally, nanocellulose can enhance the mechanical properties of various polymer matrices.^13^

Nanocellulose can be produced in various forms, including cellulose nanofibrils (CNFs), cellulose nanocrystals (CNCs), or cellulose nanospheres (CNSs).^16^ CNCs are characterized by elongated crystalline rods with a compact, ordered structure, resulting in less elasticity compared to CNFs.^17^ CNFs consist of alternating crystalline and amorphous regions, of bundles of cellulose chains bound into long, flexible, tangled nanofibers ranging from 1 to 100 nm in length.^3,18,19^ CNSs have a spherical morphology and nanometer size, and their spherical polymeric nanoparticles offer numerous advantages, making them suitable for applications in drug delivery, disease detection, and diagnosis within the biomedical field.^16^ A key challenge for the broader application of nanocellulose lies in developing sustainable and economically viable production techniques.^20^

The most common technique for preparing nanocellulose is acid hydrolysis, with sulfuric acid being the most used.^11,21^ However, this method has several drawbacks: the acid corrodes equipment, poses environmental and health hazards, requires large amounts of water, and has a high cost.^22−24^ As an emerging method, enzymatic hydrolysis may be an interesting process for nanocellulose production. An emerging alternative, enzymatic hydrolysis, offers a potentially more environmentally friendly and sustainable approach to nanocellulose production compared to acid hydrolysis.^25^ Enzymes have high substrate specificity, targeting specific lignocellulosic linkages. Furthermore, enzymatic hydrolysis is conducted in mild thermal and pressure conditions, making it less energy-intensive and generating less hazardous waste.^26,8^ Additionally, it minimizes the presence of chemical compounds that could interfere with subsequent processes using the carbohydrate-rich fraction.^27,8^

To implement the biorefinery concept, various raw materials have already been utilized to produce bioethanol and other bioproducts. For instance, cashew apple bagasse (CAB), an agroindustrial waste, is composed of cellulose (18–21% w/w), hemicellulose (10–19% w/w), and lignin (35–43% w/w).^28,29^ This residue has been evaluated as a support for enzymatic immobilization^30^ and to produce ethanol,^9,31^ xylitol,^32,33^ and nanocomposites composed of lignin and Fe_3_O_4._^34^ Additionally, nanocellulose can be extracted from this waste as part of these integrated processes.

In this context, this study aims to obtain and characterize nanocelluloses from CAB through enzymatic hydrolysis. Initially, the cashew apple bagasse was pretreated with different methods to evaluate the influence of this step on the structure of the nanocellulose obtained. Also, this study aims to assess the resulting nanostructures for their application in Bioplastic production, focusing on their characterization, biodegradability, and mechanical properties.

## 2 Materials and Methods

### 2.1 Lignocellulosic Material

The CAB from *Anacardium occidentale* L. species was kindly donated by the Brazilian Food and Beverage Company S/A – Industry EBBA, located in Ceará, Brazil. The CAB was washed with distilled water, dried at 60 °C for 24 h, and milled. Particles with a size between 0.25 mm and 0.84 mm were selected for pretreatments.

### 2.2 Pretreatments of CAB

Two different pretreatments, alkaline and acid-alkali, were carried out on CAB. The alkaline pretreatment (PTA) was performed using a 1.25 mol/L sodium hydroxide (NaOH) solution and 10% (w/v) CAB and conducted at 28 °C and 150 rpm for 4 h. Afterward, the solid fraction was separated by filtration, washed with distilled water until reaching pH 7.0, dried, and labeled CAB-PTA.^11^

The acid-alkali pretreatment (PT-HA) was conducted in two stages according to the methodology proposed by Rocha et al.^35^ The first stage was carried out at 121 °C for 30 min using 0.6 mol/L H_2_SO_4_ and 20% w/v CAB. In the second stage, the solids from the first-stage pretreatment were added to 1.0 mol/L NaOH at a solid fraction of 7.5% (w/v) and pretreated at 121 °C for 30 min. The pretreated solid was recovered by vacuum filtration, washed with distilled water until a pH of 7.0 was reached, dried, and labeled CAB-PT-HA.

### 2.3 Chemical Characterization and X-ray Diffractogram of CAB and Pretreated CAB

Compositional analysis of CAB and pretreated CAB (CAB-PTA and CAB-PT-HA) was performed following NREL analytical procedures (NREL/TP, 510-42619 Series).^36^ The total solid content was determined using the NREL procedure TP-510-42621,^37^ and the structural analyses of carbohydrates and lignin were conducted according to the NREL procedure TP510-42618.^38^

X-ray diffraction was performed to analyze the crystal structure of untreated and pretreated CAB using Xpert MPD (PANalytical) equipment with CoKα radiation (λ = 1,7889 Å), operating at 40 kV and 40 mA. Measurements were obtained with an angular step (2θ) of 0.013°, time per step of 68.85 s (speed of 0.049°/s), and within a 2θ range between 10° and 100°. Moreover, the crystallinity index (CI) was calculated using Segal’s empirical method.^39^

### 2.4 Obtaining of Nanocellulose

Nanocellulose was produced using a sequential enzymatic hydrolysis and ultrasonic process from pretreated CAB (CAB-PTA or CAB-PT-HA) based on the methodology proposed by Meyabadi and Dadashian^40^ and Meyabadi et al.,^41^ with some modifications. Enzymatic hydrolysis was carried out using 5 g/L cellulose from pretreated CAB in a 50 mM sodium citrate buffer (pH = 4.8) and using an enzymatic load of 7.5 FPU/g_cellulose_ or 12 FPU/g_cellulose_ of cellulase enzyme from *Trichoderma reesei* (Sigma-Aldrich). The bioprocesses were conducted at 50 °C and 200 rpm for 24 h. To prevent microbial growth during enzymatic hydrolysis, 40 μL of tetracycline (10 mg/mL in 70% v/v ethanol) was added. As a result of the enzymatic hydrolysis, a gelatinous suspension was obtained, which was centrifuged at 5000 rpm for 20 min.

The liquid phase (rich in sugars) was analyzed by high-performance liquid chromatography, and the gelatin suspension was suspended in distilled water. The aqueous dispersions containing the nanocellulose were then sonicated using an ultrasonic tip (power 500 W) at 40% amplitude for 15 min with 5 s pulses, followed by a mechanical disperser (Ultra-Turrax T10 BASIC) at 15,000 rpm for 15 min. The materials obtained were labeled NC-HE-A (nanocellulose from CAB-PTA) and NC-HE-HA (nanocellulose from CAB-PT-HA). All experiments were performed in triplicate.

To perform yield calculations, the material resulting from the resuspension was dried on an infrared scale (Mars-ID2000) at 105 °C, in triplicate, to obtain the dry mass. Using this value, it was possible to calculate the yields of the obtained nanocellulose. The nanocellulose yield was calculated relative to the pretreated CAB mass (*Y*_NC_) according to eq 1, where *Y*_NC_ represents the yield relative to the pretreated biomass, *m*_NC_ is the nanocellulose mass, and *m*_initial_ is the mass of pretreated biomass used in hydrolysis (CAB-PTA or CAB-PT-HA).
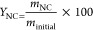
1

### 2.5 Preparation of Films

The films were prepared using the methodology developed by Yuan and Chen.^42^ To prepare the control film (CF), a starch solution (2.5% w/v) was dissolved in distilled water at 70 °C with continuous stirring to achieve a uniformly dispersed suspension. Glycerin (1.3%, w/v) was then added to the starch solution and heated at 90 °C for 30 min. For standardization, the mixture was kept at 75 °C with magnetic agitation for an additional hour. Approximately 54 mL of the starch-glycerin suspension was then cast into a polytetrafluoroethylene mold (diameter = 4 cm) and dried at 45 °C for 24 h in an oven, resulting in the control starch-based film.

To enhance the properties of the starch film, different percentages (5 and 7% w/v) of nanocellulose obtained via enzymatic hydrolysis using CAB-PT-HA as a precursor (NC-HE-HA) were added. The starch and glycerin suspension, after heating at 90 °C for 30 min, was cooled, and NC-HE-HA (at 5% or 7% w/v) was incorporated into the mixture. The mixture was then agitated with a magnetic stirrer at 75 °C for 1 h. Approximately 54 mL of the resulting suspension was cast into a poly(tetrafluoroethylene) mold with a diameter of 4 cm and dried at 45 °C for 24 h in an oven. The starch and nanocellulose films were successfully prepared and dried in an oven at 40 °C for 24 h. The resulting films were named F5-NC-HA (containing 5% w/v NC-HE-HA) and F7-NC-HA (containing 7% w/v NC-HE-HA).

### 2.6 Characterization of Nanostructures

Fourier transform infrared (FTIR) spectroscopy with attenuated total reflection (ATR) was used to analyze the functional groups on the surface of nanocellulose and the films. The samples were analyzed directly in a Cary 630 spectrometer (Agilent Technologies) over the range of 4000–650 cm^–1^, with an average of 32 scans and a spectral resolution of 1 cm^–1^.

The thickness of the films was measured with an absolute AOS digital pachymeter (Mitutoyo). The microstructural study of the nanocelluloses was conducted by using atomic force microscopy (AFM) and scanning electron microscopy (SEM). AFM analysis was performed with a Bruker model MM8, using the quantitative nanomechanics (QMN) scanning mode. The samples were deposited on mica previously cleaved with a glass capillary and left to dry until a film of nanoparticles was formed. The analyses were performed using cantilevers with a nominal spring constant of 0.4 N/m and a nominal tip radius of 2 nm with a scan resolution of 256 × 256 samples per line and a frequency of 0.5 Hz. For SEM analysis, the samples were deposited on carbon tape, coated, and metalized with 20 nm of gold by using a QT150 ES metallizer (Quorum). Then, SEM images were captured with a Quanta 450 FEG-FEI instrument using a 20 kV incident electron beam.

The zeta potential was measured in triplicate to assess the stability of the resuspension resulting from enzymatic hydrolysis (potential nanocellulose) using Malvern 3000 Zetasizer Nano ZS equipment (Malvern instruments). Samples were diluted 10-fold in deionized water, and measurements were performed at 25 °C.

Thermal stability of the nanocellulose samples (10 mg) was evaluated by thermogravimetric analysis (TGA) using a PerkinElmer STA 6000 simultaneous thermal analyzer. The analysis was conducted over the range of 30–500 °C with a heating rate of 10 °C min^–1^ under an inert nitrogen atmosphere at a flow rate of 50 mL min^–1^.

### 2.7 Nanomechanical Analysis Using AFM

Initially, the films were cut into squares, measuring 13 mm on each side, and fixed to the equipment’s sample holder (magnetic disk) of the AFM using double-sided adhesive tape. The AFM experiments were conducted with a Multimode 8 (Bruker, Santa Barbara) using Nanoscope Analysis 2.0 software (Bruker) in the PeakForce QNM mode.^43^ A cantilever with a spring constant of 0.4 N/m and a nominal tip radius of 2 nm was employed with a scan resolution of 256 × 256 lines and a scan frequency of 0.5 Hz.

For nanomechanical analysis, approximately 196,000 force curves were collected for each 5 μm map in three different regions of each film, using four different indentation frequencies: 2, 1, 0.5, and 0.25 kHz, analyzing YM and energy dissipation of the samples at varying frequencies.^44^ YM was obtained using the Derjaguin–Muller–Toporov (DMT) model, which describes the interaction between two spheres, modeled with an undeformed sphere in contact with a rigid plane.^45^ The contact area depends on parameters of the indenter (AFM probes) and the sample surface (stiffness). The load force applied between the surfaces is directly influenced by this contact, as described by eq 2.
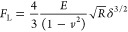
2where *R* is the delay radius, δ is the indentation, *E* is Young’s modulus, and v is Poisson's ratio of the film’s surface.

Dissipation data were obtained from the energy loss due to the nonlinearity of the interaction between the tip and the sample through higher harmonic modes.^46^ This quantity is calculated by eq 3.
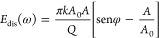
3where *k* is the spring constant, *Q* is the quality factor, and *A* is the cantilever oscillation amplitude. Pure elastic deformation, for example, corresponds to very low dissipation values.

### 2.8 Analysis of Water-Holding Capacity and Water Vapor Permeability of Biofilms

The water-holding capacity (WHC) of films was obtained according to the method developed by Khurshid et al.^47^ Films were cut with dimensions of 2.5 × 2.5 cm, weighed (*W*_1_), and submerged in distilled water for 2 min, with the experiment performed in triplicate. After the films were removed from the water, the final weight (*W*_2_) was measured after excess water had been removed. Then, the films’ WHC (%) was calculated using eq 4.
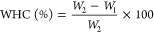
4

The experiment to determine the water vapor permeability (WVP) of films was performed following the method described by Cazón et al.^48^ A cup with an area of 1.25 × 10^–3^ m^2^ was filled with 60 mL of distilled water, leaving a headspace of less than 50 mm between the water surface and the film. The cup was sealed with O-rings to avoid water evaporation through the edges. Then, it was placed in a stove (Tecnal TE-397/4, Piracicaba, SP, Brazil), containing silica at the bottom for moisture control, and maintained at 30 °C. The system was conditioned for at least 3 h to ensure it was at the selected temperature at the beginning of the experiment.

The films were then placed over the cup, covering the entire opening, and the system was incubated in an oven at 30 °C for 24 h. Samples were taken every hour and weighed, and the value was recorded.

The water vapor transmission rate (WVTR), water vapor permeance, and WVP were calculated according to eqs 5–7, respectively:
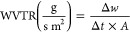
5
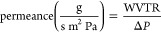
6
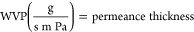
7where Δ*w*/Δ*t* (g/s) is the flux measured as the weight loss of the cell per unit of time and calculated as the slope of the weight loss of the cup versus time; *A* (m^2^) is the actual exposed area determined by the mouth cup diameter; and Δ*P* (Pa) is the water vapor pressure differential, calculated as 4245 Pa at 30 °C^48,49^ assuming a full water vapor saturation in the headspace and a full-dried environment provided by the silica. Each experiment was performed in triplicate.

### 2.9 Evaluation of Biodegradability of Biofilms

The biodegradability of the films was investigated for a period of 35 days using the soil burial method, as described by Reshmy et al.^1^ and Reshmy et al.^4^ The soil used was acquired from a local flower shop in Fortaleza city (Ceará, Brazil).

Films measuring 1 × 1 cm (*W*_1_) were placed in a container filled with the soil and buried at a depth of 10 cm. Regular observations were made by periodically removing the films, washing and drying them in an oven at 70 °C for 24 h, and then weighing them (*W*_2_). The percentage of weight loss (*W*_L_) was calculated by using eq 8.
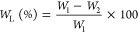
8

### 2.10 Statistical Analysis

The data were analyzed using OriginPro 9.0 software, employing analysis of variance followed by Tukey’s test. The significance of differences between groups was determined based on *p*-values, with the statistical significance set at a 5% level (*p* < 0.05).

## 3 Results and Discussion

### 3.1 Raw Material and the Influence of Pretreatment on CAB Composition

The CAB was composed of 15.4 ± 3.2% w/w cellulose, 9.6 ± 1.2% w/w hemicellulose, and 46.6 ± 2.2% w/w lignin and ash, with a CI of 54.40%. Before enzymatic hydrolysis, it is necessary to pretreat the CAB to make the cellulose more accessible to enzymatic action by removing hemicellulose and lignin. Therefore, acid-alkali and alkaline pretreatments were performed to enhance the efficiency of enzymatic hydrolysis and evaluate the influence of these pretreatments on the production and properties of nanocellulose.

The composition of pretreated CAB varied with the type of pretreatment carried out. The CAB obtained from acid-alkali pretreatment (CAB-PT-HA) was composed of 58.8% w/w cellulose, 5.6% w/w hemicellulose, and 12.0% w/w lignin. This material’s CI was 62.8%, higher than the CI of untreated CAB (54.4%) due to the removal of hemicellulose and lignin. In the acid pretreatment, diluted sulfuric acid solubilized the hemicellulose fraction and facilitated lignin removal during the subsequent alkaline pretreatment. Sodium hydroxide used in the final step of pretreatment caused swelling of the lignocellulosic matrix and cleaved the aryl ether linkages in lignin, promoting its restructuring and removal. Consequently, this process also dissolved the amorphous fraction, thereby affecting the crystallinity of the material.^9,50−52^

The pretreated CAB obtained through a single-step alkaline pretreatment (PTA), named CAB-PTA, had a composition of 30.3% w/w cellulose, 8.0% w/w hemicellulose, and 26.6% w/w lignin. Although CAB-PTA had a higher lignin content than CAB-PT-HA, it exhibited a higher CI (62.8%), with a difference of 6.8% between the CIs of these materials. According to Alvira et al.^53^ and Rocha et al.,^51^ NaOH causes swelling in lignocellulosic materials, expanding their area and reducing the degree of polymerization. As a consequence, the bonds between lignin and carbohydrates are broken, mainly in the amorphous regions, increasing the crystallinity.

**Figure 1:**
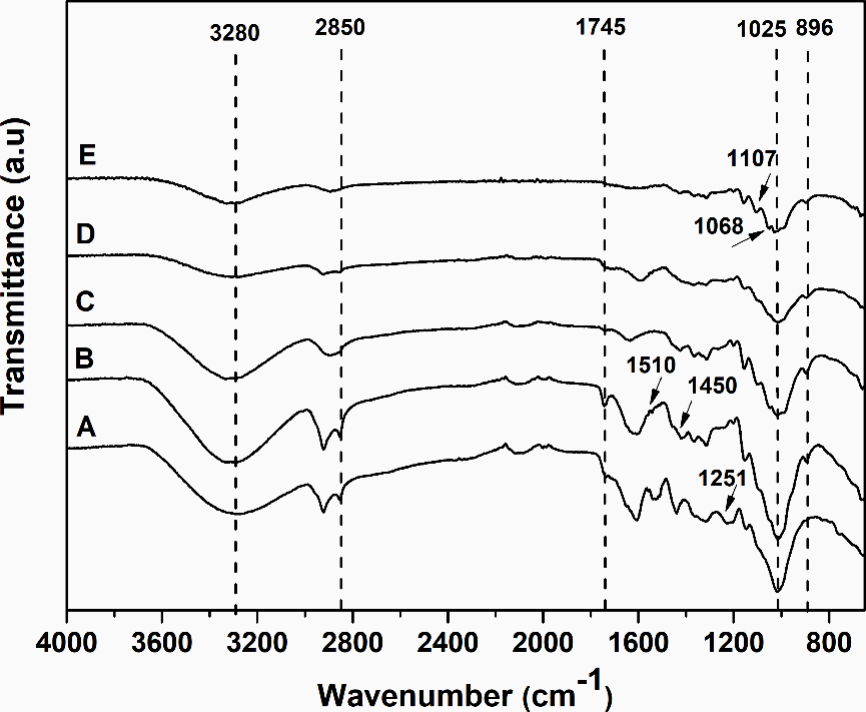
FTIR spectra of (A) untreated CAB, (B) CAB-PTA, (C) CAB-PT-HA, (D) NC-HE-A, and (E) NC-HE-HA.

The chemical composition analyses are corroborated with FTIR analyses. The spectra of CAB, CAB-PTA, and CAB-PT-HA can be seen in Figure 1, along with the spectra of the respective nanoparticles derived from these materials, discussed in Section 3.2.1. The spectra showed two main absorbance regions, in the ranges of 700–1800 and 2700–3500 cm^–1^.

The CAB spectrum (Figure 1A) shows a band at 1025 cm^–1^, representing the vibration in the C–O stretching of the cellulose molecule, which is also present in the CAB-PTA and CAB-PT-HA spectra. Additionally, the CAB spectrum features bands at 1155 cm^–1^, representing the C–O (an asymmetric bridge that extends in ester bonds); bands between 1450 and 1510 cm^–1^, referring to the conjugated elongation of C=O in aromatic rings, characteristic of lignin^29^; a band at 1745 cm^–1^, corresponding to the ester-linked acetyl, ferulic, and p-coumaric groups present in hemicellulose and lignin structures^54^; and an absorption band at 1251 cm^–1^, attributed to the C–O stretching of acetyl groups present in hemicellulose molecular chains.^29^

The changes promoted by alkaline and acid-alkali pretreatments in the structure of CAB are visible in the FTIR spectra of CAB-PT-HA and CAB-PTA. In these spectra, a band at 896 cm^–1^ is identified, corresponding to the C–H stretching of β-glycosidic bonds between the glucose units of cellulose. In the CAB-PT-HA spectrum, the band at 1745 cm^–1^, which corresponds to the ester groups linked to acetyl, ferulic, and p-coumaric referring to hemicellulose and lignin, is not observed (Figure 1C), indicating the removal of the hemicellulose and lignin during the acid-alkali pretreatment. However, the band at 1745 cm^–1^ is still present in the CAB-PTA spectrum, indicating the presence of lignin in CAB-PTA (Figure 1B).

### 3.2 Production and Characterization of Nanocelluloses

In the processes for obtaining nanocellulose, two enzymatic loads of the cellulolytic complex were evaluated (7.5 or 12 FPU/g_cellulose_). However, nanocellulose was not produced in the process using the highest enzymatic load. Therefore, the results presented below refer to the enzymatic processes using 7.5 FPU/g cellulose. The higher enzymatic load did not favor nanocellulose obtaining because it increased the efficiency of cellulose hydrolysis, resulting in higher glucose concentration in the hydrolysate.

#### 3.2.1 Chemical Structure Analysis of Nanocelluloses

The alkaline and acid–alkali pretreatments promoted changes in the structure of the CAB as well as obtaining nanocelluloses by enzymatic hydrolysis. The modifications can be seen in Figure 1.

The modifications in the material after sequential enzymatic hydrolysis with an ultrasonic process to obtain nanocellulose were mainly observed in bands that represent the C–OH skeletal vibration, characteristic of carbohydrate molecules in the cellulose structure (band at 1107 cm^–1^, Figure 1D,E). This band was more defined in the spectra of CAB-PTA and CAB-PT-HA (pretreated CAB) shown in Figure 1B,C, respectively, indicating a reduction of cellulose present in the structure after the process of obtaining the nanocellulose (Figure 1D,E). In the spectra of the NC-HE-A and NC-HE-HA nanocelluloses, a band at 1068 cm^–1^ was identified, which is associated with ether binding (C–O–C) of the skeletal vibration of pentose and hexose,^55^ constituents of hemicellulose and cellulose chains, respectively. Bands at 1107, 1150, and 1161 cm^–1^, attributed to the glycosidic structure,^56^ were also observed in the spectra of the NC-HE-A and NC-HE-HA nanocelluloses (Figure 1D,E, respectively).

#### 3.2.2 Morphology and Particle Size Analysis of Nanocelluloses

To analyze the morphology and size of the particles, the solid fractions resulting from the sequential enzymatic hydrolysis combined with the ultrasonic process were analyzed by using AFM.

**Figure 2:**
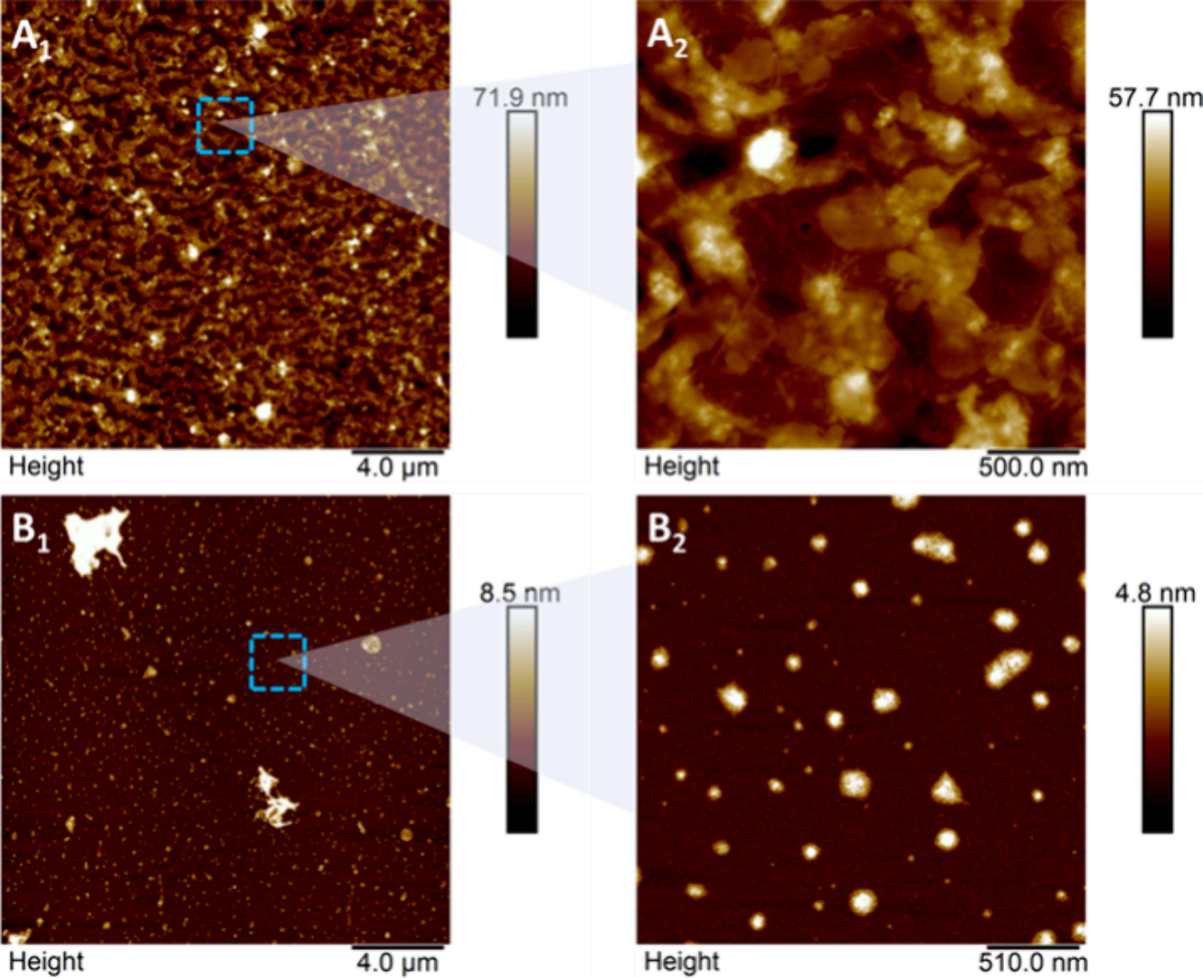
Atomic force micrographs of nanocellulose particles obtained by sequential enzymatic hydrolysis combined with an ultrasonic process using CAB-PTA (NC-HE-A, panels A1 and A2) and CAB-PT-HA (NC-HE-HA, panels B1 and B2) as precursors.

Figure 2 displays micrographs of the structures of the particles obtained from CAB-PTA or CAB-PT-HA through sequential enzymatic hydrolysis using the ultrasonic process. This allows for observation of structural differences, indicating that the choice of precursor material affects the resulting nanocellulose.

In the micrographs (Figure 2A_1_,A_2_) of particles obtained from CAB-PTA, an agglomerated structure is observed, which hinders the identification of the formation of nanoparticles. CAB-PTA, containing 26.6% w/w lignin, impedes enzymatic hydrolysis by forming spatial barriers due to the lignin content in the fiber, caused by the lignin content present in the fiber. This hinders the interaction between the cellulase enzyme complex and the cellulose in the fiber, making it difficult to form Nanostructures.^57^

In contrast, the micrographs (Figure 2B_1_,B_2_) of particles obtained from CAB-PT-HA reveal well-defined spherical Nanostructures, or nanospheres, with diameters ranging from 57.26 to 220.66 nm. The material used as a precursor (CAB-PT-HA) had a lower lignin content (12.0 ± 0.1% w/w) and a higher cellulose content (58.8 ± 1.2% w/w) compared to CAB-PTA. The reduced lignin content and higher cellulose content in CAB-PT-HA facilitate greater exposure of cellulose to enzymatic attack, enhancing hydrolysis efficiency and enabling the successful production of nanocellulose.

The evaluation of the process and materials in this study enabled the production of spherical nanocellulose, which has a higher specific surface area than nanofibrils and nanocrystals of similar size. This increased surface area improves the dispersion of the nanocellulose within the polymer matrix and promotes the formation of more intermolecular interactions.^58,59^ These interactions restrict the mobility of the polymer chains, enhancing the thermal stability of the composite material. Furthermore, the stronger bonding between the spherical nanocellulose and the polymer matrix contributes to the overall improvement in both mechanical and thermal properties of the nanocomposites.^60^

Cellulose nanoparticles with a spherical morphology were also obtained through the enzymatic hydrolysis of cotton fiber waste (cellulose content >95%), followed by the ultrasound process.^41^ Also, Li et al.^61^ used a two-step process (enzymatic treatment followed by high-pressure homogenization) to produce CNSs from bamboo pulp. However, these materials are more expensive than CAB.

The production of nanocellulose by enzymatic hydrolysis requires a relatively low capital investment. However, due to its initially low reaction yield, the production cost of enzymatic CNCs remains high, which makes it less commercially attractive. To make enzymatic CNCs competitive, it is crucial to improve the yield of enzymatic hydrolysis.^62^

Thus, this study achieved a yield of CNSs (NC-HE-HA) of 65.1 ± 2.9%, which could contribute to reducing the overall production costs of nanocellulose via enzymatic hydrolysis. Improved yields can enhance the economic feasibility of this process, particularly as the cost of enzymes continues to decrease with ongoing research and process optimization. Furthermore, the enzymatic hydrolysis process produced a stream with a high concentration of sugars (glucose and cellobiose), which can be converted into valuable byproducts such as 1,2-butanediol, lactic acid, or squalene.^62^

The yield of CNSs (NC-HE-HA) obtained in this study was higher than that reported by Meyabadi et al.,^41^ who obtained nanoparticles from waste cotton fibers (washed with nonionic surfactant (1 g/L Irgasol) for 1 h, rinsed with distilled water, and oven-dried at 105 °C for 3 h) through enzymatic hydrolysis using 2.3% v/v cellulase enzyme and 5 g/L cotton for 175 h, resulting in a yield of less than 20%. Meyabadi et al.^41^ attributed the low yield to the enzyme converting a significant amount of cellulose into glucose, cellobiose, cellotriose, and cellotetraose. The higher yield of nanoparticles obtained in this study is likely due to the shorter reaction time (24 h) compared to the 175 h used by Meyabadi et al.,^41^ as well as the enzymatic load.

However, Li et al.^61^ investigated the production of nanospheres (CNS) from bamboo fibers through a continuous three-step process, which included enzymatic pretreatment, high-pressure homogenization, and enzymatic hydrolysis (conducted for 6 h with an enzymatic load of 10 FPU/g_cellulose_ from a cellulase complex, which contains endogluconases, exogluconases, and β-1,4-gluconases), and they reported a yield of 74%. This comparison highlights that the material, process, and operating conditions significantly influence the production of nanocellulose.

The liquid fractions obtained during the enzymatic hydrolysis of CAB-PTA and CAB-PT-HA contained low concentrations of cellobiose and glucose, indicating low cellulose digestibility. The CAB-PTA hydrolysate obtained after 24 h of reaction contained 0.98 g/L cellobiose and 0.70 g/L glucose (Figure S1), while the CAB-PT-HA hydrolysate contained 1.52 g/L cellobiose and 0.80 g/L glucose (Figure S2).

In both hydrolysates, the cellobiose concentration was higher than the glucose concentration due to the low activity of β-glucosidase in the enzymatic complex. Therefore, to obtain a higher glucose concentration, it is necessary to add this enzyme to hydrolyze cellobiose to glucose. Some authors^9,63,64^ added β-glucosidase enzyme to the enzymatic hydrolysis of CAB to enhance cellulose conversion into glucose. However, this supplementation negatively impacted the production of nanocellulose; then, β-glucosidase was not added in the processes for obtaining nanocellulose from CAB in this study.

Analyzing the results from this study alongside those reported in the literature, it is evident that the choice of enzymes, enzymatic load, and reaction time are crucial factors influencing the performance of Nanostructures obtained through enzymatic hydrolysis. Also, the composition of the pretreated material plays a significant role; in this study, nanocellulose could not be obtained using CAB-PTA as a precursor under the evaluated conditions.

Nanocellulose (NA-HE-HA) obtained from CAB-PT-HA showed a negative zeta potential (−30 mV) at the examined pH (pH 6.8 ± 0.2). The zeta potential measures the magnitude of attractive or repulsive forces between particles, making it an important tool for predicting and describing the colloidal behavior of nanocellulose suspensions.

According to Bhattacharjee,^65^ particles with zeta potential values of ±0 – 10, ±10 – 20, ±20 – 30, and > ±30 mV are classified as highly unstable, relatively stable, moderately stable, and highly stable, respectively. Uniform dispersion of nanocellulose is crucial for enhancing the mechanical properties of the final nanocomposite products.^66^ Therefore, NA-HE-HA demonstrates the stability necessary for applications that require mechanical strength, which will be further discussed in Section 3.4.3.

### 3.3 Thermal Analysis

**Figure 3:**
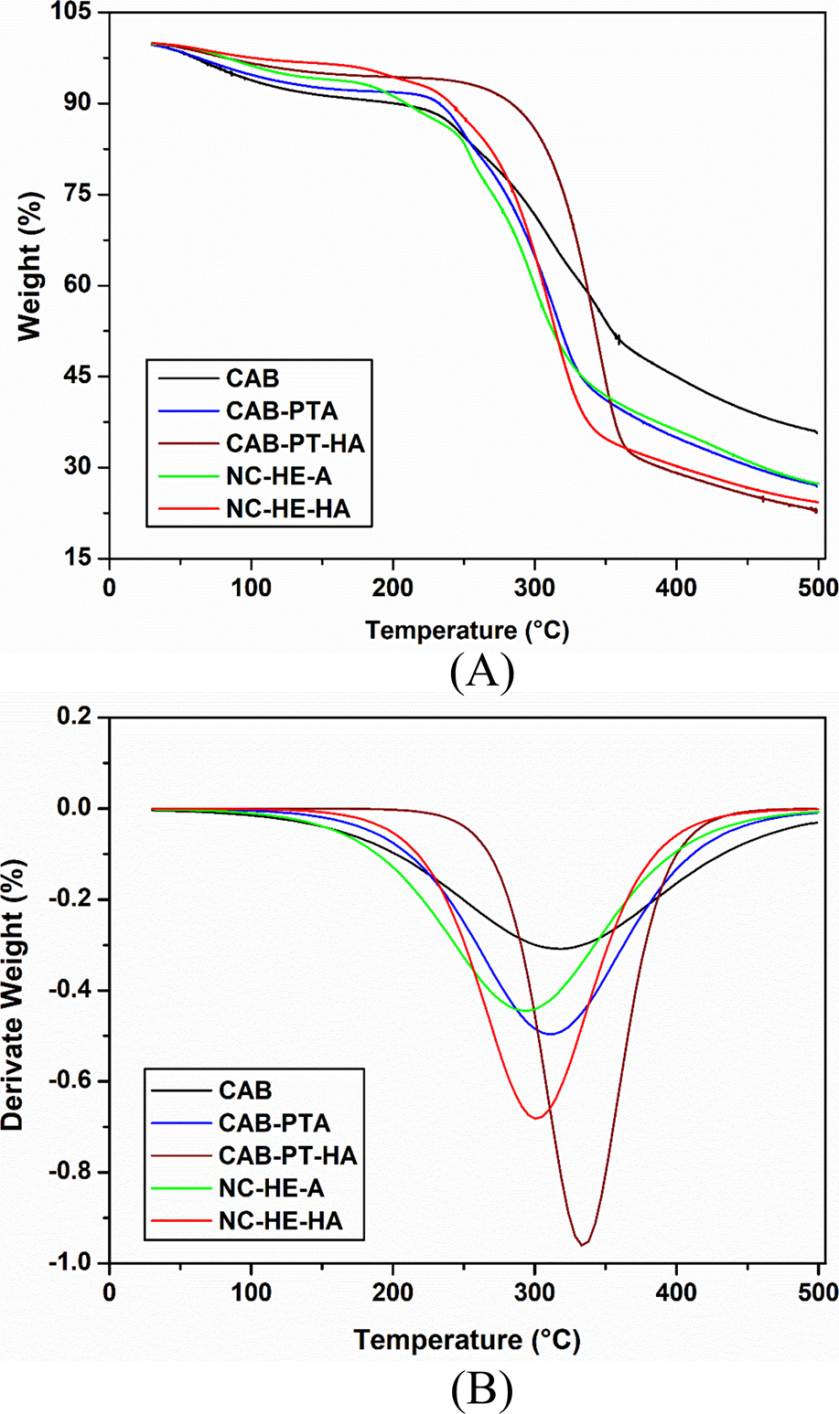
Thermogravimetric curves of the untreated CAB, CAB pretreated with alkaline pretreatment (CAB-PTA), CAB pretreated with acid-alkali pretreatment (CAB-PT-HA), and materials obtained by sequential enzymatic hydrolysis combined with ultrasonic processing (NC-HE-A and NC-HE-HA). (A) TGA and (B) DTG.

The thermogravimetric curves of untreated CAB, pretreated CABs (CAB-PTA and CAB-PT-HA), and the nanoparticles resulting from the sequential enzymatic hydrolysis method combined with ultrasonic processing are presented in Figure 3.

The thermogravimetric curves of CAB (Figure 3) exhibit a mass loss due to water evaporation at approximately 100 °C, followed by a significant mass loss between 215 °C that increases up to 350 °C, which can be attributed to the decomposition of hemicellulose, a peak degradation rate observed around 315 °C. Comparing the curves for CAB and CAB-PTA (CAB subjected to alkaline pretreatment), it is evident that the decomposition rate of CAB-PTA is faster between 250 and 300 °C, in comparison to CAB. The alkaline pretreatment led to the removal of a small amount of lignin, while the hemicellulose content remained relatively unchanged.

However, the material resulting from the acid-alkali pretreatment (CAB-PT-HA) demonstrated greater resistance to thermal degradation, with a maximum degradation temperature around 340 °C. This enhanced thermal stability is probably due to its higher cellulose content in CAB-PT-HA compared to CAB and CAB-PTA, along with its high CI (62.8%). According to Meyabadi et al.,^41^ cellulose fibers that are more ordered require more energy for polymer degradation.

In CAB-PT-HA, cellulose is more exposed and accessible to enzymes than in CAB-PTA, leading to more efficient nanostructure production. However, careful control is necessary to prevent excessive cellulose digestibility.

NC-HE-HA nanocellulose exhibited high thermal stability, although lower than its precursor, CAB-PT-HA. The difference in the thermograms of these materials is the most noticeable between 200 and 350 °C, corresponding to the degradation temperature range of hemicellulose and cellulose. According to Meyabadi et al. and Michelin et al.,^27^ the decrease in the thermal stability of NC-HE-HA may be linked to the reduction in particle size, which increases the surface area exposed to heat. Moreover, the presence of lignin and hemicellulose in the CAB-PT-HA precursor could contribute to its increased stability during thermal degradation compared to NC-HE-HA.

NC-HE-HA showed a slightly lower thermal stability compared to NC-HE-A. This can be attributed to differences in the structure and morphology of the materials, which arise from the distinct compositions of their respective precursors. NC-HE-A did not display a cellulose nanostructure, as identified in the AFM analysis (Figure 2A_1_,A_2_), where an agglomerated structure is observed. The precursor of NC-HE-A, CAB-PTA, contains greater amounts of lignin and hemicellulose compared to the CAB-PT-HA precursor of NC-HE-HA. These components, particularly lignin, may help protect the cellulose structure during the nanostructure formation process, contributing to the better thermal stability of NC-HE-A, but making the formation of Nanostructures difficult.

NC-HE-HA nanocellulose showed greater thermal stability than spherical nanocellulose obtained by acid hydrolysis from CAB (also pretreated by acid-alkaline pretreatment) in the experiments carried out by Araújo et al.^11^ Furthermore, Lu and Hsieh^67^ reported that spherical nanocrystals prepared by sulfuric acid hydrolysis begin decomposing around 150 °C. In contrast, NC-HE-HA decomposed starting around 200 °C and goes up to 340 °C, with a maximum degradation at 300 °C.

Thus, the lower thermal stability of the nanoparticles prepared by sulfuric acid hydrolysis suggests a different decomposition mechanism. It was reported that introducing sulfate groups during sulfuric acid hydrolysis significantly reduces the activation energies for cellulose nanoparticle degradation.^67,41^

Nanostructures produced by enzymatic hydrolysis have a high number of free hydroxyl groups on their surface, which can strongly interact through hydrogen bonds and van der Waals forces. This interaction can result in low colloidal stability for Nanostructures produced by acid hydrolysis but greater thermal stability.^68,27^ However, the nanocellulose obtained in this study (NC-HE-HA) demonstrated both colloidal and thermal stability, which is interesting (beneficial) for applying this nanomaterial.

Thermal stability is a crucial property for Nanostructures, requiring processing at temperatures above 200 °C, such as the manufacturing of most composite materials.^69,8^

### 3.4 Production and Characterization of Films

Starch-based films have the potential to replace petroleum-based polymeric films in many applications, especially in food handling and packaging. These films extend the shelf life of foods by acting as a barrier or controlling the permeability of water, gases, and volatile compounds.^70^ Starch-based materials also exhibit advantages in biocompatibility and biodegradability, making them suitable for medicinal drug delivery, packaging, and agricultural applications.^71^ Therefore, it is essential to develop edible and biodegradable particles to reinforce and improve the mechanical properties of starch-based films. Various mixing and compounding techniques are being researched and developed, including the incorporation of cellulosic fibers at the nanometer scale (Ali et al.^70^; Li et al.^72^). Thus, this study evaluated the use of NC-HE-HA nanocellulose, obtained via sequential enzymatic hydrolysis with an ultrasonic process, in the production of films. Enzymatic methods for the synthesis of Nanostructures offer several benefits, such as avoiding the use of corrosive agents, thereby reducing potential toxicity to the human body and allowing for more widespread use in food, cosmetics, and pharmaceuticals.^13^

#### 3.4.1 Chemical Structure Analysis of Films

**Figure 4:**
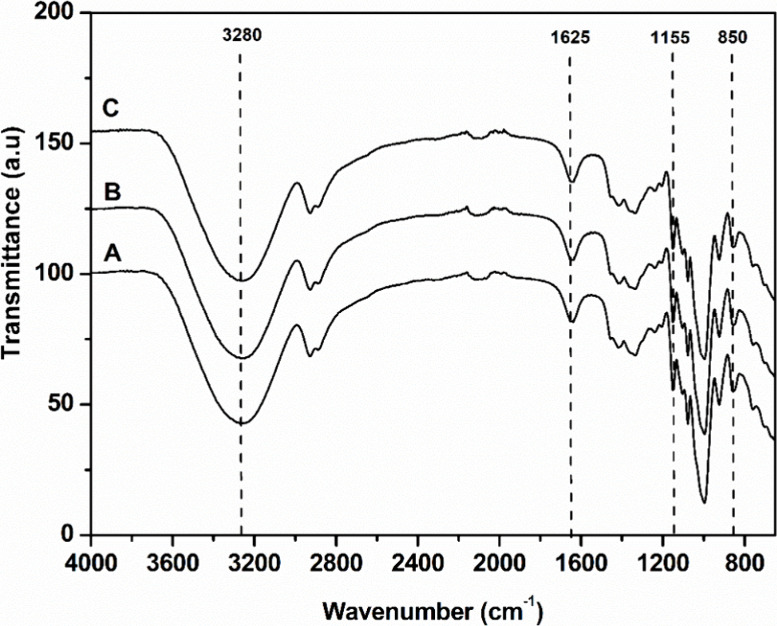
Spectroscopy with attenuated total FTIR spectra of the (A) CF, (B) film containing 5% w/v NC-HE-HA (F5-NC-HA), and (C) film containing 7% w/v NC-HE-HA (F7-NC-HA).

Figure 4 shows the FTIR spectra of the CF and films containing 5% w/v (F5-NC-HA) and 7% w/v (F7-NC-HA) of NC-HE-HA nanocellulose.

The CF spectrum, composed of glycerol and starch, is shown in Figure 4A. The spectra for F5-NC-HA (Figure 4B) and F7-NC-HA (Figure 4C) films were identical to the spectrum of the CF, indicating that bands did not appear or disappear after the addition of Nanostructures (NC-HE-HA). A possible explanation is that starch and cellulose have very similar chemical structures. According to Builders and Arhewoh,^73^ starch consists of two high-molecular-weight polymers, amylose and amylopectin, both of which are composed of D-glucose units. Amylose is linked by α-1,4 bonds and amylopectin by α-1,6 glycosidic bonds, whereas cellulose is a linear homopolymer of glucose units linked by β-1,4-glycosidic bonds.^74^ The main difference between starch and cellulose is the type of glycosidic bonds (α-glycosidic in starch and β-glycosidic in cellulose), with both having the same functional groups. Thus, with the FTIR technique, identifying the insertion of nanocellulose becomes quite challenging. However, AFM analyses were performed and are discussed in the following sections.

#### 3.4.2 Morphology Analysis of Films

**Figure 5:**
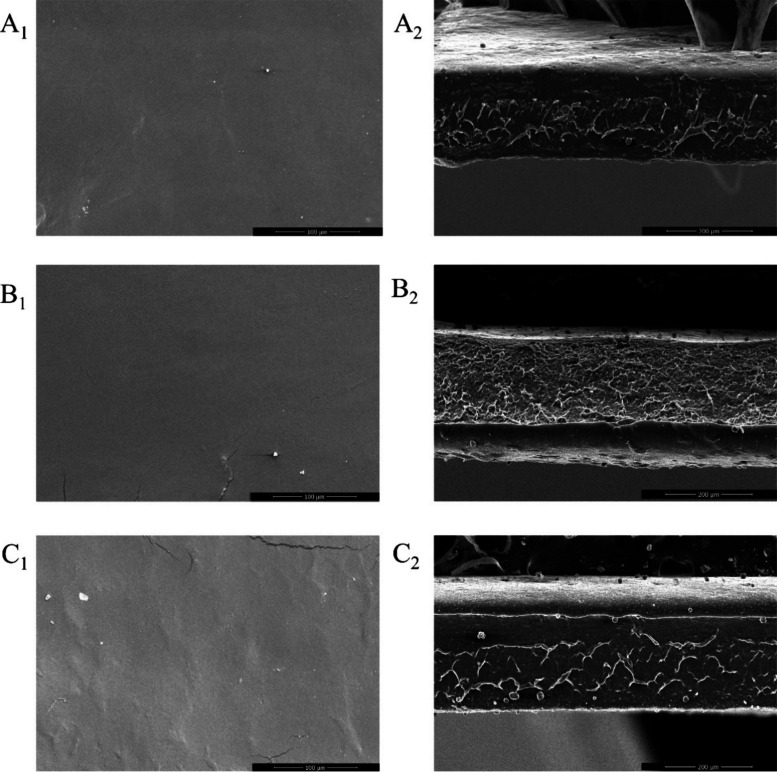
SEM micrographs of the surface (images numbered 1) and thickness (images numbered 2) of the films: (A_1_,A_2_) control; (B_1_,B_2_) F5-NC-HA; and (C_1_,C_2_) F7-NC-HA.

Figure 5 shows the micrographs of the CF and the films obtained using different percentages of NC-HE-HA (5 or 7% w/v).

The CF (Figure 5A), composed only of starch and glycerol, exhibited a very smooth and uniform surface. Similar characteristics were observed in the films incorporating 5% w/w NC-HE-HA (F5-NC-HA, Figure 5B) or 7% w/w NC-HE-HA (F7-NC-HA, Figure 5C) nanostructures. In these films, the interface between the matrix and the particles appeared smooth and homogeneous, with no spaces between them, indicating good material compatibility.^70^

The thicknesses of the control, F5-NC-HA, and F7-NC-HA films were 0.30 ± 0.02, 0.32 ± 0.01, and 0.31 ± 0.03 mm, respectively, showing basically no difference, which is important for comparing film properties.

#### 3.4.3 Ultrastructural Analysis

**Figure 6:**
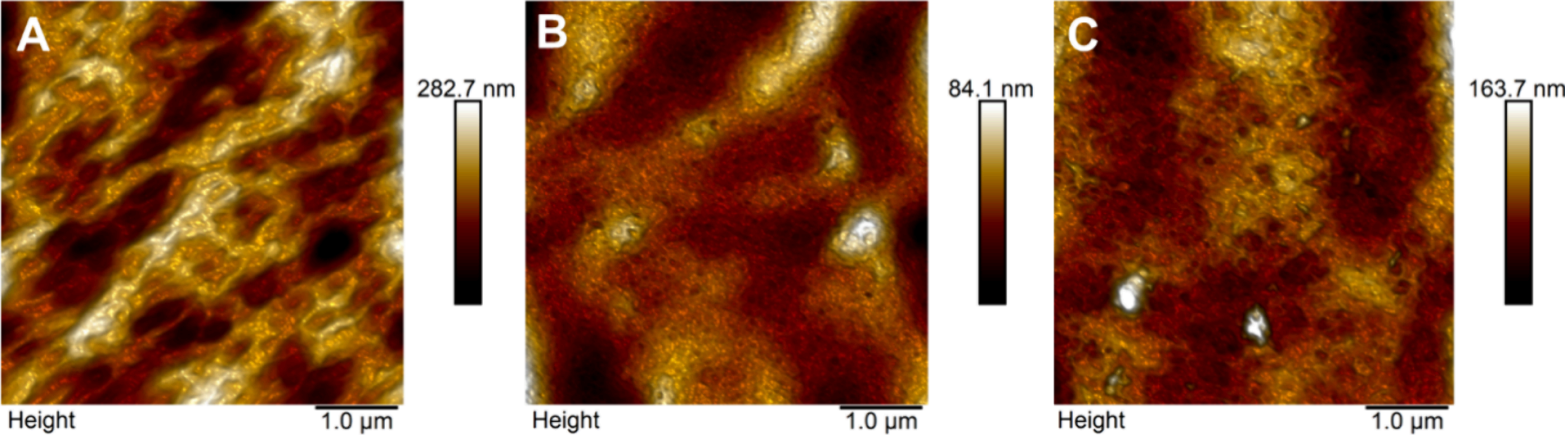
AFM topographic maps. (A) CF, (B) F5-NC-HA film, and (C) F7-NC-HA film.

Figure 6 shows representative topographic images of the synthesized films. The nanocelluloses alter the three-dimensional morphology of the films, making the surfaces more uniform after functionalization with NC-HA-HE. The mean square roughness values of the control (Figure 6A), F5-NC-HA (Figure 6B), and F7-NC-HA (Figure 6C) films were 41.1 nm, 11.1 nm, and 20.8 nm, respectively.

#### 3.4.4 Nanomechanical Properties

**Figure 7:**
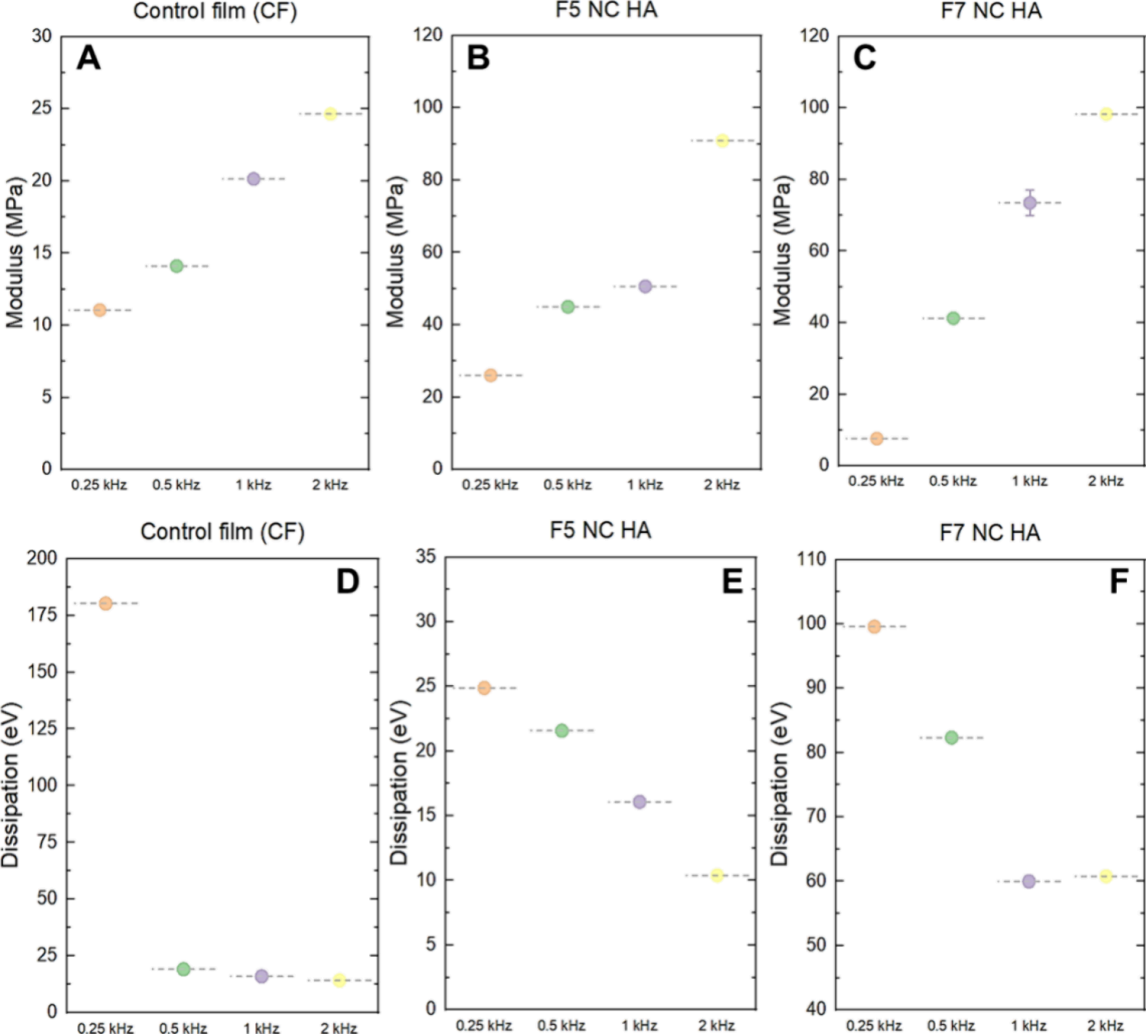
Nanomechanical properties were determined by AFM. YM values of the (A) control, (B) F5-NC-HA, and (C) F7-NC-HA films. Energy dissipation of the (D) control, (E) F5-NC-HA, and (F) F7-NC-HA films.

Young’s modulus (YM) and energy dissipation data were analyzed for the control sample (CF) and films incorporated with NC-HE-HA (F5-NC-HA and F7-NC-HA), showing their differences and highlighting the viscoelastic behavior for each oscillation frequency (Figure 7).

For all samples, an increase in the YM was observed with an increase in the cantilever oscillation frequency (Figure 7A–C). After film functionalization, there was a tendency for the YM values of the nanostructured films to increase (Figure 7B,C), indicating that the mechanical resistance of these films can be improved with the addition of nanoparticles. The energy dissipation values for all samples (Figure 7D–F) tended to decrease with increasing oscillation frequency. After film functionalization (Figure 7E,F), the F5-NC-HA films showed decreased energy dissipation at the nanoscale. The viscoelastic character of pure films (CF) changed after functionalization with nanoparticles, resulting in a reduced dissipative component and an increased elastic component, which can provide greater resistance.

#### 3.4.5 WHC

The WHC is an important property for determining the applicability of films, especially concerning their biodegradability. Films with higher WHC can be more susceptible to degradation when exposed to high-moisture foods.^47^ Then, the WHC of the synthesized films was measured, and the results are presented in Table 1.

**Table 1:** WHC of the Control, F5-NC-HA, and F7-NC-HA Films

FILMS	WHC (%)cc
control	37.26 ± 0.33
F5-NC-HA	34.80 ± 0.63
F7-NC-HA	33.67 ± 0.35

The films incorporating different percentages of NC-HE-HA nanocellulose exhibited a lower WHC compared with the CF. Specifically, the CF exhibited a WHC of 37.26 ± 0.33%, while the F5-NC-HA and F7-NC-HA films showed WHC values of 34.80 ± 0.63 and 33.67 ± 0.35%, respectively, a difference of approximately 3%. These results suggest that at the concentrations tested (5 and 7%), nanocellulose did not improve the water retention of the CFs. In fact, the slight reduction in WHC can be attributed to the decreased roughness of the films, as evidenced by AFM analyses, which may reduce the available surface area for water absorption.

As noted by Reshmy et al.,^4^ high WHC can compromise the integrity of films when in contact with high-moisture foods as it may cause disruption of bonds within the structure. Therefore, the incorporation of nanocellulose could improve the mechanical stability of the films, making them more suitable for packaging applications, where control of moisture is important.

Vargas et al.^75^ produced films from starch and glycerol, incorporating various percentages of red rice flour and starch (*Oryza glaberrima*) into the polymeric matrix, which demonstrated potential for food packaging. These obtained films exhibited WHC values ranging from 49.5 to 51.9%, higher than those observed in this study. Consequently, the films developed here present a promising alternative in terms of shelf life and biodegradability, especially given their composition of nontoxic starch and cellulose, making them suitable for food packaging.

#### 3.4.6 WVP

WVP measures the amount of water vapor that diffuses through a film per unit area, time, and pressure gradient. Table S1 presents the WVP data for the synthesized films.

The WVP values for the control and F5-NC-HE and F7-NC-HE films were 1.83 × 10^–9^ ± 3.26 × 10^–9^, 1.32 × 10^–9^ ± 3.23 × 10^–10^, and 1.39 × 10^–9^ ± 1.20 × 10^–10^ g s^–1^ m^–1^ Pa^–1^, respectively. The incorporation of nanocellulose reduced the WVP. Two processes allow gases and vapor to pass through polymeric materials: (1) the polymer’s morphology, such as the pores or cracks; and (2) the solubility–diffusion effect due to the combination of Fick’s law of diffusion and Henry’s law of gas solubility.^48^ Then, based on process (1), the films functionalized with nanocellulose exhibited a lower roughness and likely fewer pores, thus reducing the vapor permeability.

In food packaging, materials are expected to minimize or prevent moisture transfer between the food and the external environment, preserving the food’s water content.^76,77^ Therefore, a low WVP is desirable, making the results of this study promising. The incorporation of nanocellulose achieved a lower WVP, enhancing the material’s attractiveness for food packaging.

#### 3.4.7 Evaluation of Biodegradability

**Figure 8:**
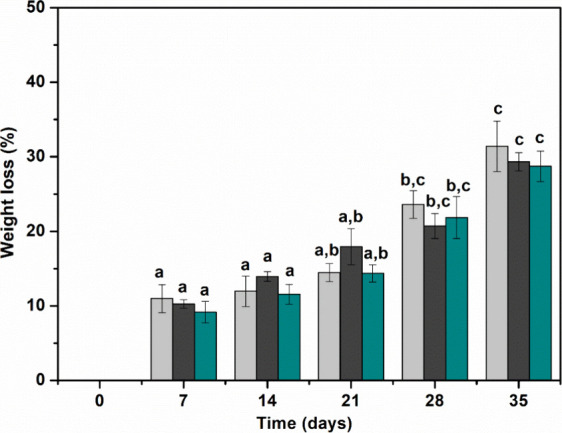
Percentage of weight loss for the control (light-gray bar), F5-NC-HA (dark-gray bar), and F7-NC-HA (dark-cyan bar) films over 35 days. Different superscripts (a–c) indicate significant differences (*p* < 0.05).

The biodegradability of films was assessed using the soil burial method for 35 days, with weight loss results depicted in Figure 8. The biodegradation test was performed using a method that has been previously applied to similar biofilms. The conditions for the test were adapted for this study and included a specific temperature (28 C), humidity (2.55%), and soil characteristics (low organic content).

The degradation of the films increased over time, showing a significant difference in mass loss (Figure 8). After 7 days of soil burial under laboratory conditions, the biodegradation percentage was approximately 10%, rising to 30% with 35 days of assay. Films containing nanocellulose showed a similar biodegradation percentage to the CF, with no significant difference at a 95% confidence level.

The soil used in our experiment might not have supported high microbial activity due to its composition and organic matter content. Additionally, the temperature and humidity conditions could have influenced the microbial degradation rates. The soil used in this experiment was very sandy with a moisture content of 2.55 ± 0.05% w/w. This low moisture content, coupled with the lack of other factors that favor polymer degradation, such as microbial activity, wind, rainfall, and temperature variation,^78^ may have affected the biodegradation process and the time frame of 35 days.

Teramoto et al.^79^ studied the biodegradability of composites of aliphatic polyesters (poly(e-caprolactone), poly(3-hydroxybutyrate-*co*-3-hydroxyvalerate) (PHBV), poly(butylene succinate) (PBS), and poly(lactic acid) (PLA)) with 10% by weight of untreated and acetic anhydride-treated abaca fibers by soil burial for 180 days. Pure PHBV exhibited the lowest biodegradability with only 29% weight loss, followed by PHBV/AA–abaca with about 48% weight loss and untreated PHBV/abaca showed the highest biodegradability, with significant fragmentation after 60 days, with the degradation period greater than that performed in this study. Therefore, the films synthesized in this study demonstrated greater biodegradability within a shorter period compared to films proposed by Teramoto et al.^79^

Altaee et al.^80^ investigated the biodegradation of polyhydroxybutyrate (PHB) and titanium oxide composites (PHB-TiO_2_) in soil with pH 7.30 and 80% humidity at 30 °C. PHB-TiO_2_ showed a 51% weight loss after 3 weeks, and PHB alone had a 62% weight loss. In some of the mentioned studies, total degradation of the films occurred after more than 35 days. The films that degraded in a shorter time were subjected to burial in soil with higher humidity than that of the soil used in this study, indicating that these factors significantly impact the total biodegradation process.

In summary, the films incorporating varying percentages of nanocellulose exhibited biodegradable properties, although a longer period is required for complete decomposition.

Among the methods used for producing CNSs, techniques such as acid hydrolysis,^59^ enzymatic treatments,^81^ and chemical oxidation^82^ are noteworthy. However, acid hydrolysis presents some drawbacks, including high water consumption for residual acid removal, lower thermal stability compared to the starting cellulosic material, and limitations in functionalization, which hinder its application in various large-scale industrial sectors.^83^ In contrast, this study employed enzymatic hydrolysis using the *Trichoderma reesei* cellulase enzyme complex for 24 h, resulting in CNSs with diameters ranging from 57.26 to 220.66 nm and a yield of 65.1 ± 2.9%. This yield is higher than that reported by Dias et al.,^81^ who achieved a yield of 41% using bleached eucalyptus Kraft pulp and the endoglucanase enzyme (FiberCare, Novozymes). Another study by Chen et al.,^84^ also using bleached Kraft eucalyptus pulp and *Aspergillus niger* cellulase, obtained a yield of only 13.6%.

## 4 Conclusions

The nanocellulose in a spherical shape was successfully obtained from acid-alkali-pretreated CAB using sequential enzymatic hydrolysis followed by ultrasonic processing, achieving a high yield. This method represents a sustainable alternative to the conventional chemical processes. The resulting nanostructures exhibited good thermal and colloidal stability and can be used as polymeric reinforcement agents in biofilm production. The films incorporated with nanocellulose were synthesized using components from natural and nontoxic resources, and they showed a lower WHC and lower WVP compared to the CF while maintaining the same level of biodegradability. AFM analyses indicated that incorporating nanoparticles into the films enhances their nanoscale mechanical properties. These characteristics suggest that these biofilms have promising potential for applications in food packaging.

## Supporting Information Available

Determination of carbohydrates in the liquid fraction resulting from enzymatic hydrolysis and analysis of WVP of the films (PDF)
