# Supplementary material for: Production of Cellulose Nanoparticles from Cashew Apple Bagasse by Sequential Enzymatic Hydrolysis with an Ultrasonic Process and Its Application in Biofilm Packaging
Source: ACS Omega. 2024 Dec 13;9(51):50671–84. doi: 10.1021/acsomega.4c08702 (PMC11683648; doi:10.1021/acsomega.4c08702)
Supplement: Supplementary file 1 — ao4c08702_si_001.pdf [file ao4c08702_si_001.pdf]

Production of cellulose nanoparticles from cashew apple bagasse by enzymatic hydrolysis sequential at the ultrasonic process and its application in biofilms packaging

[a] Federal University of Ceará, Department of Chemical Engineering, Bioengineering and Biomass Valorization Laboratory, Fortaleza, Ceará, Brazil

[b] State University of Acaraú Valley, Exact Sciences and Technology Center, Chemistry Course, Sobral, Ceará, Brazil

[c] Federal University of Maranhão, Department of Physics, Laboratory of Biophysics and Nanosystems, São Luís, Maranhão, Brazil

[d] Embrapa Tropical Agroindustry, Rua Dra Sara Mesquita 2270, Planalto do Pici, CEP 60511-110 Fortaleza, CE, Brazil

***Corresponding author**: Maria Valderez Ponte Rocha
Federal University of Ceará,
Department of Chemical Engineering
Bioengineering and Biomass Valorization Laboratory
Campus do Pici, Bloco 709, Fortaleza, Ceará, Brazil
Zip code: 60455-760
phone: +55 85 3366-9611; fax: +55 85 3366-9610.
e-mail: valderez.rocha@ufc.br; valponterocha@yahoo.com.br

Supplementary material

In this supplementary material there are tables and figures that were discussed in the article following the order given by the same.

Determination of carbohydrates in the liquid fraction resulting from enzymatic hydrolysis

After the enzymatic hydrolysis, the liquid fraction was analyzed to determine the concentrations of carbohydrates (cellobiose and glucose) by HPLC using a system equipped with a refractive index detector (Water, Milford, MA, USA). The samples (20 μL) were analyzed using an Aminex HPX-87H column (Bio-Rad, Hercules, CA, USA) at 65 °C, and eluted with 5 mmol. L^{-1} H_2SO_4 aqueous solution at a flow rate of 0.5 $mL.min^{-1}$. The results were reported in the Fig. S1 and S2.

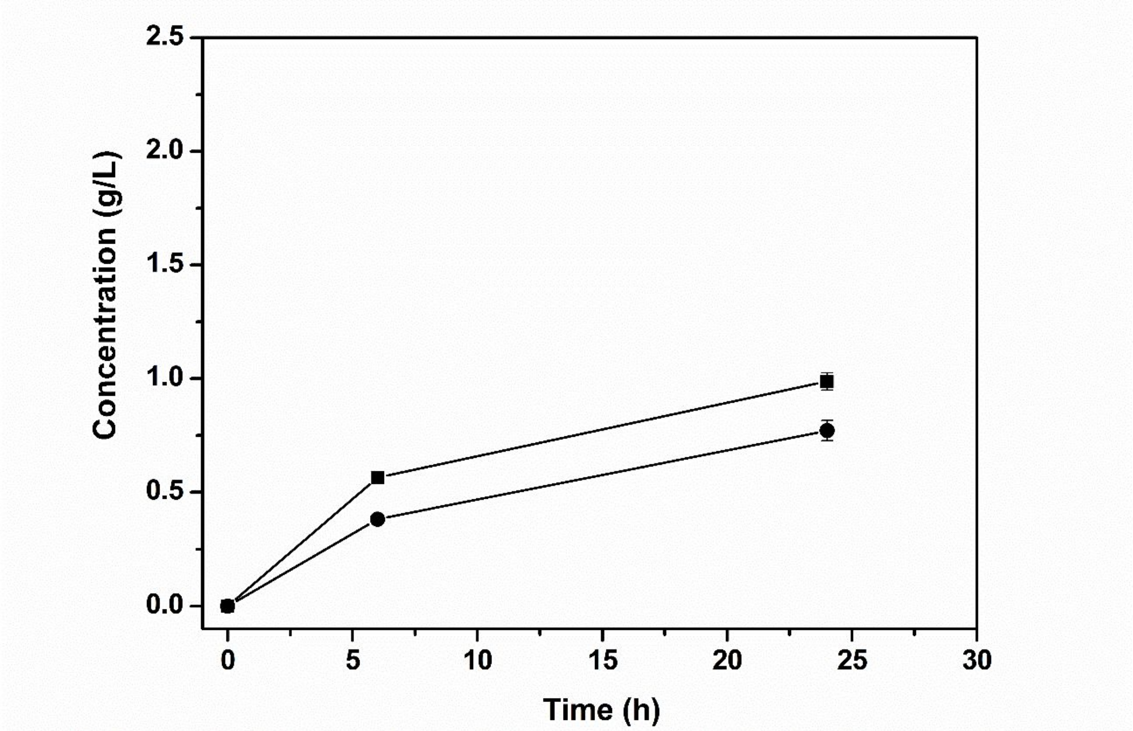

Figure S1. profiles of cellobiose and glucose concentrations resulting from the enzymatic hydrolysis of CAB-PTA, using the cellulase enzyme complex from *Trichoderma reesei* under constant agitation of 200 RPM at 50 °C and 24 h with a load of 7.5 $FPU/g_{cellulose}$ (■) cellobiose e (●) glucose.

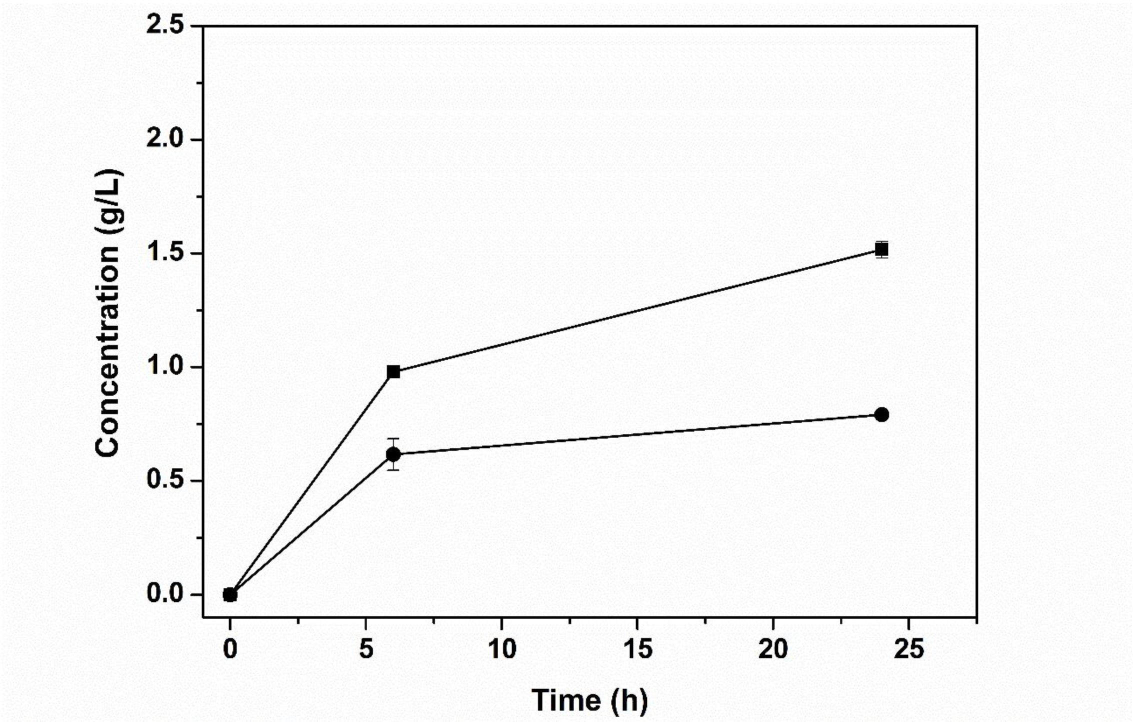

Figure S2. Profiles of cellobiose and glucose concentrations resulting from the enzymatic hydrolysis of CAB-PT-HA, using the cellulase enzyme complex from *Trichoderma reesei* under constant agitation of 200 RPM at 50 °C and 24 h with a load of 7.5 FPU/g $_{\text{cellulose}}$ (■) cellobiose e (●) glucose.

Analysis of Water Vapor Permeability (WVP) of the films

Table S1. The results obtained in water vapor permeability (WVP) analysis at 30 °C of with a pressure difference (ΔP) of 4245 Pa, and the films area of 0.001256 m^2. The values were calculated using Equations 5, 6 and 7.

**Samples**		**CF_1**	**CF_2**	**CF_3**
**Slop**	$g.day^{-1}$	2.813	2.875	1.847
**R^2**		0.952	0.957	0.952
**Thickness**	M	0.0003	0.00030	0.00028
**WVTR**	$g.day^{-1}.m^{-2}$	2239.79	2289.15	1470.19
**Permeance**	$g.\ day^{-1}.m^{-2}.Pa^{-1}$	0.5276	0.5393	0.3463
**WVP**	$g.day^{-1}.m^{-1}.Pa^{-1}$	0.000158289	0.000159081	9.52423×10^{-5}
**WVP**	$g.s^{-1}.m^{-1}.Pa^{-1}$	1.83205×10^{-9}	1.84122×10^{-9}	1.10234×10^{-9}
**Average WVP**		1.83205×10^{-9}		
**Error**		3.26352×10^{-10}		
**Samples**		**F5-NC-HE_1**	**F5-NC-HE_2**	**F5-NC-HE_3**
**Slop**	$g.day^{-1}$	3.125	2.061	2.169
**R^2**		0.944	0.956	0.957
**Thickness**	m	0.0003	0.00030	0.00028
**WVTR**	$g.day^{-1}.m^{-2}$	2487.82	1640.66	1726.96
**Permeance**	$g.\ day^{-1}.m^{-2}.Pa^{-1}$	0.5861	0.3865	0.4068
**WVP**	$g.day^{-1}.m^{-1}.Pa^{-1}$	0.000175818	0.000114015	0.000111876
**WVP**	$g.s^{-1}.m^{-1}.Pa^{-1}$	2.03493×10^{-9}	1.31962×10^{-9}	1.29486×10^{-9}
**Average WVP**		1.31962×10^{-9}		
**Error**		3.23418×10^{-10}		
**Samples**		**F7-NC-HE_1**	**F7-NC-HE_2**	**F7-NC-HE_3**
**Slop**	$g.day^{-1}$	2.137	2.448	2.026
**R^2**		0.953	0.956	0.955
**Thickness**	m	0.0003	0.00030	0.00028
**WVTR**	$g.day^{-1}.m^{-2}$	1701.70	1948.82	1613.34
**Permeance**	$g.\ day^{-1}.m^{-2}.Pa^{-1}$	0.4009	0.4591	0.3801
**WVP**	$g.day^{-1}.m^{-1}.Pa^{-1}$	0.000120261	0.00013543	0.000104515
**WVP**	$g.s^{-1}.m^{-1}.Pa^{-1}$	1.39191×10^{-9}	1.56748×10^{-9}	1.20967×10^{-9}
**Average WVP**		1.39191×10^{-9}		
**Error**		1.20013×10^{-10}		
